# Group 13 exchange and transborylation in catalysis

**DOI:** 10.3762/bjoc.19.28

**Published:** 2023-03-21

**Authors:** Dominic R Willcox, Stephen P Thomas

**Affiliations:** 1 EaStCHEM School of Chemistry, University of Edinburgh, Edinburgh, EH9 3FJ, United Kingdom

**Keywords:** catalysis, group 13 exchange, hydroboration, main group, transborylation

## Abstract

Catalysis is dominated by the use of rare and potentially toxic transition metals. The main group offers a potentially sustainable alternative for catalysis, due to the generally higher abundance and lower toxicity of these elements. Group 13 elements have a rich catalogue of stoichiometric addition reactions to unsaturated bonds but cannot undergo the redox chemistry which underpins transition-metal catalysis. Group 13 exchange reactions transfer one or more groups from one group 13 element to another, through σ-bond metathesis; where boron is both of the group 13 elements, this is termed transborylation. These redox-neutral processes are increasingly being used to render traditionally stoichiometric group 13-mediated processes catalytic and develop new catalytic processes, examples of which are the focus of this review.

## Introduction

**Scheme 1:**
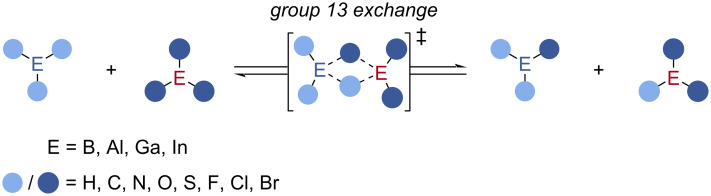
Group 13 exchange.

Group 13 compounds have found widespread use in stoichiometric organic transformations, typically in the functionalisation of unsaturated bonds [1–5], and, more recently, frustrated Lewis pair (FLP) chemistries including small molecule activations and C–H insertion reactions [6–10]. Group 13 exchange is the transfer of one or more substituents from one group 13 element to another group 13 element by σ-bond metathesis, a redox neutral process (Scheme 1). Stoichiometric group 13 exchange reactions are key to the synthesis of group 13 reagents including in organoboron chemistry [11–28], and more recently with aluminium [29–39], gallium [36,40–44], and indium [36,45] reagents being used for the preparation of group 13 reagents. Group 13 exchange has recently been used to enable catalytic turnover in traditionally stoichiometric reactions, expanding the use of group 13 compounds in catalysis beyond their typical use as Lewis acids [46]. This strategy has allowed the synthesis of bench-stable boronic ester products, rather than sensitive alkylboranes, and enabled the use of substoichiometric amounts of enantioenriched boron reagents, which can be challenging to prepare. This review will explore the use of group 13 exchange reactions as a general method for catalytic turnover, and serves to expand on the previously published review on transborylation-enabled boron catalysis to include a broader range of catalysts and turnover reagents [47].

## Review

### Boron catalysis

**Scheme 2:**
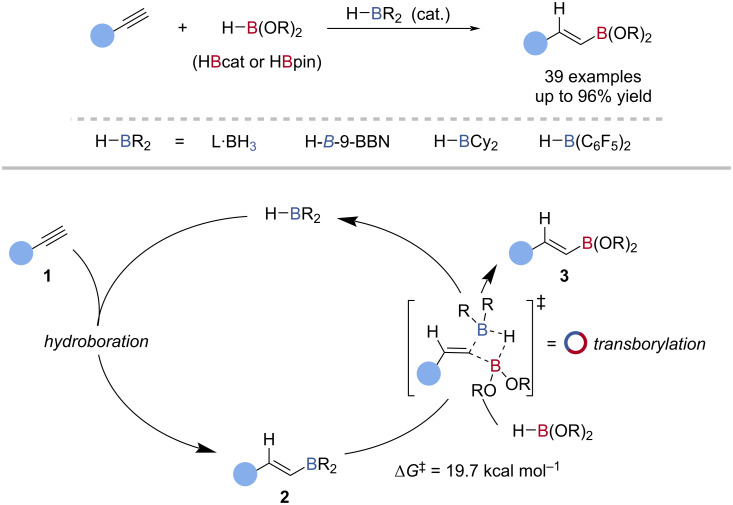
Borane-catalysed hydroboration of alkynes and the proposed mechanism.

The borane-catalysed hydroboration of alkynes was first reported by Periasamy using *N*,*N*-diethylaniline·BH_3_ as the catalyst and HBcat as the turnover reagent (terminal reductant) [48–49]. This was followed by Hoshi who used dialkylboranes, 9-borabicyclo(3.3.1)nonane (H-*B*-9-BBN) and dicyclohexylborane (Cy_2_BH) to catalyse the hydroboration of alkynes with HBcat [50]. Hoshi later reported that Cy_2_BH [51] and in situ generated bis(pentafluorophenyl)borane, Piers’ borane [52], catalysed the hydroboration of alkynes with HBpin, to give alkenyl pinacol boronic esters. Tris(2,4,6-trifluorophenyl)borane [53], tris(3,4,5-trifluorophenyl)borane [54], BH_3_ [55–57], and H-*B*-9-BBN [58] have also been reported as catalysts for the hydroboration of alkynes with HBpin (Scheme 2). Lloyd-Jones et al. investigated the mechanism of this reaction and found transborylation, group 13 exchange between boron atoms, enabled catalytic turnover [58]. The alkyne **1** and dialkylborane reacted to give an alkenylborane **2**. Transborylation with HBpin gave the alkenyl boronic ester **3** and regenerated the catalyst, HBR_2_. Isotopic labelling (H^10^Bpin) confirmed B–C(sp^2^)/B–H transborylation proceeded by σ-bond metathesis, and not ligand exchange. Using kinetic and computational analyses, the B‒C(sp^2^)/B‒H transborylation transition state was determined to have a free energy barrier of approximately 20 kcal mol^−1^ (Δ*G*^‡^_calc_ = 19.7 kcal mol^−1^; Δ*G*^‡^_exp_ = 20.3 kcal mol^−1^) (Scheme 2).

**Scheme 3:**
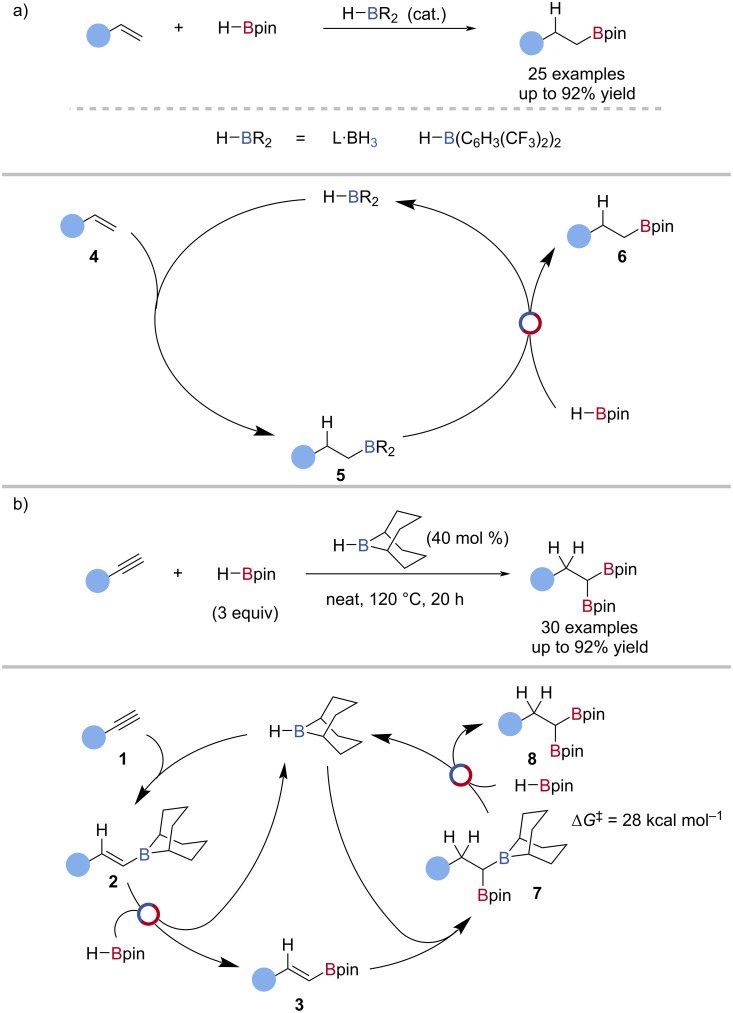
a) Borane-catalysed hydroboration of alkenes and the proposed mechanism. b) H-*B*-9-BBN-catalysed double hydroboration of alkynes and the proposed mechanism.

The borane-catalysed hydroboration of alkenes has been less explored, with tris[3,5-bis(trifluoromethyl)phenyl]borane [59], tris(3,4,5-trifluorophenyl)borane [54], and BH_3_ [55–56] found to be competent catalysts of this transformation (Scheme 3a). The mechanism was proposed to be analogous to that of borane-catalysed alkyne hydroboration; alkene **4** hydroboration, followed by transborylation with HBpin to give the alkylboronic ester **6** and regenerate the catalyst (Scheme 3a). Thomas also reported that alkynes undergo double hydroboration using H-*B*-9-BBN as the catalyst with HBpin to give *gem*-diborylalkanes **8** (Scheme 3b), and this was proposed to occur through transborylation, with an experimentally determined free energy barrier of 28 kcal mol^−1^ for the second transborylation reaction (Scheme 3b) [60].

**Scheme 4:**
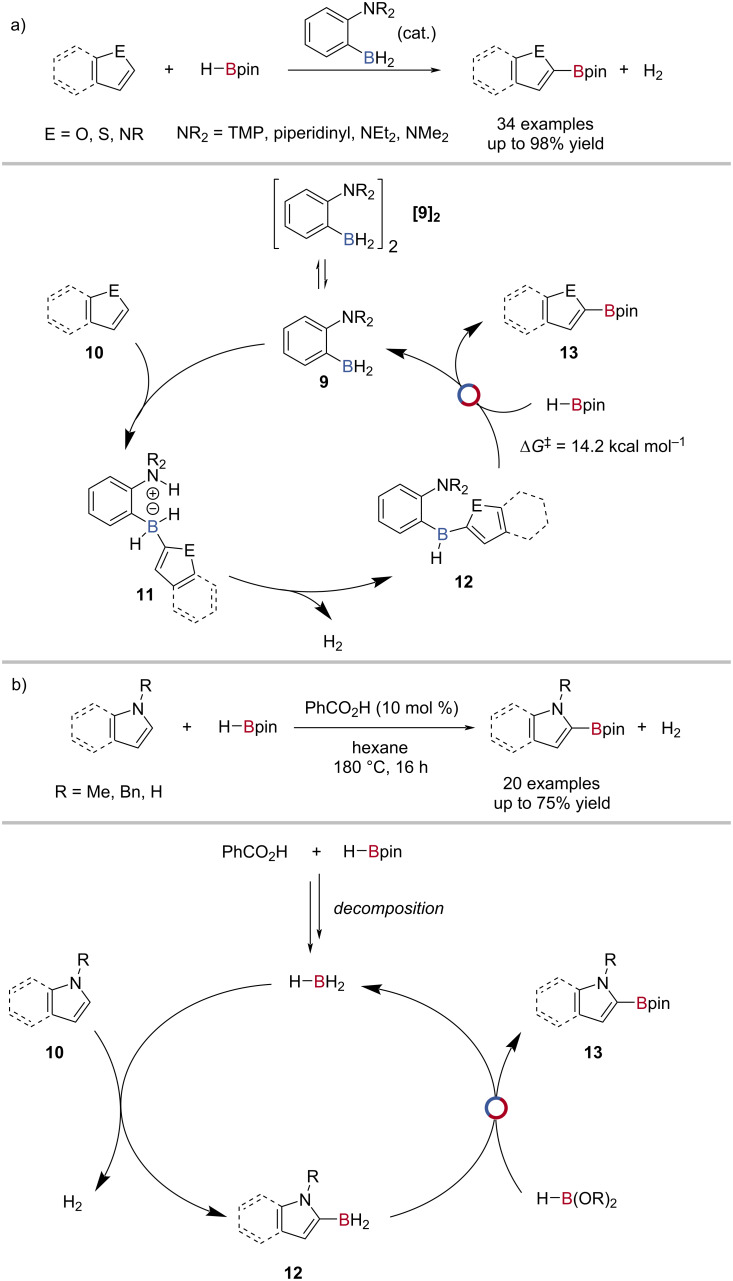
a) Amine-borane-catalysed C‒H borylation of heterocycles and the proposed mechanism. b) Benzoic acid-promoted C‒H borylation of *N*-heterocycles and the proposed mechanism, where the active catalyst BH_3_ was formed in situ from HBpin decomposition.

The seminal work from Fontaine reported that [1-(*N*-2,2,6,6-tetramethylpiperidinyl)-2-BH_2_-C_6_H_4_]_2_ catalysed the C–H borylation of heterocycles with HBpin [61], the first example of a catalytic frustrated Lewis pair (FLP)-mediated C‒H functionalisation (Scheme 4a). Using computational analysis, the mechanism of the reaction was proposed to occur by borane dimer **[9]_2_** dissociation, followed by a concerted deprotonation of the heterocycle **10** to give a zwitterionic intermediate **11**. The zwitterion then loses dihydrogen to give a neutral borane **12**, followed by B–C(sp^2^)/B–H transborylation with HBpin (Δ*G*^‡^ = 14.7 kcal mol^−1^) to give the borylated arene **13** and regenerate the catalyst (Scheme 4a). Fontaine showed that the steric bulk of the Lewis base had a significant effect on the rate of the reaction; changing the 2,2,6,6-tetramethylpiperidinyl group for a piperidinyl gave a large rate enhancement [62]. Fontaine also showed that bench-stable salts [1-(NR_2_)-2-BF_2_Y-C_6_H_4_]H (NR_2_ = 2,2,6,6-tetramethylpiperidinyl, pyridinyl, diethylamino, dimethylamino; Y = F, OMe, OH) could be used as precatalysts for the C‒H borylation, with an initial B‒Y/B‒H transborylation activating the precatalyst [62–65]. Zhang showed that benzoic acid decomposed HBpin to BH_3_ in situ to catalyse the C2‒H borylation of indoles (Scheme 4b) [66–67].

**Scheme 5:**
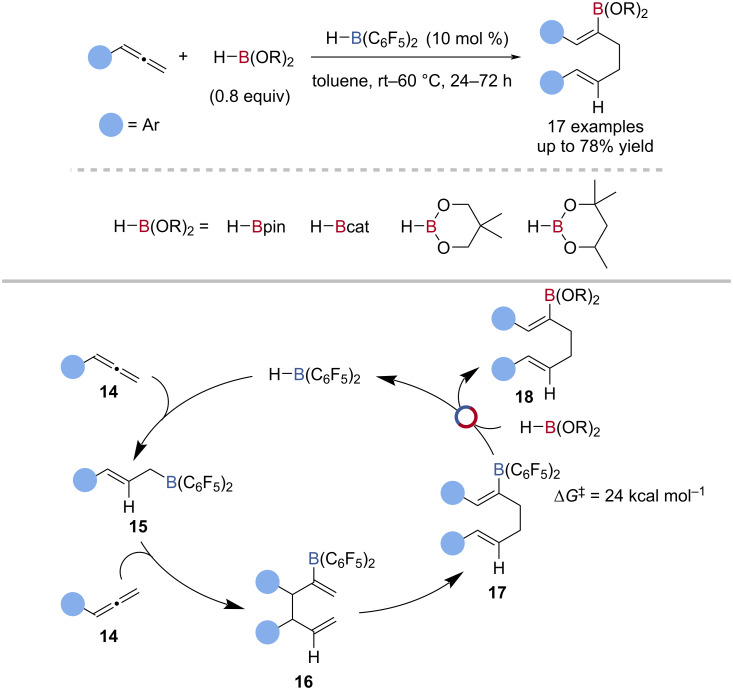
Bis(pentafluorophenyl)borane-catalysed dimerisation of allenes and the proposed mechanism.

Gellrich reported the bis(pentafluorophenyl)borane-catalysed dimerisation of allenes, using various boronates as the terminal reductant (Scheme 5) [68]. Experimental and computational studies suggested the reaction proceeded by hydroboration of the allene **14** by bis(pentafluorophenyl)borane to give an allylborane **15**, which underwent allylation of a second equivalent of the allene **14**, giving a boryl diene **16**. A Cope rearrangement of the boryl diene **16** followed by transborylation gave the dienyl boronic ester **18** and regenerated the catalyst (Scheme 5).

**Scheme 6:**
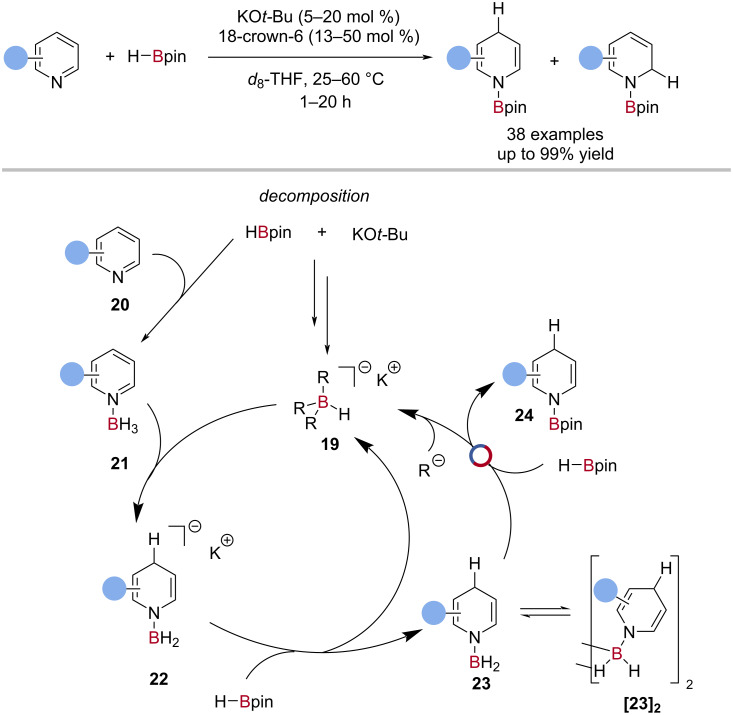
Alkoxide-promoted hydroboration of heterocycles and the proposed mechanism.

Chang reported the alkoxide-promoted hydroboration of *N*-heteroarenes with HBpin, the first explicit example of a B‒N/B‒H transborylation in catalysis (Scheme 6) [69]. Reactive intermediates were characterised and BH_3_ was observed to be generated in situ by the decomposition of HBpin. The proposed catalytic cycle involved nucleophile-promoted decomposition of HBpin to various borohydride species **19**, which reacted with the BH_3_-coordinated heterocycle **21**. Hydride transfer from the BH_3_-amide **22** to HBpin regenerated the borohydride catalyst **19**, and gave a neutral aminoborane **23**, which then underwent B‒N/B‒H transborylation with HBpin to give the *N*‒Bpin dihydropyridine **24** and BH_3_ (Scheme 6).

**Scheme 7:**
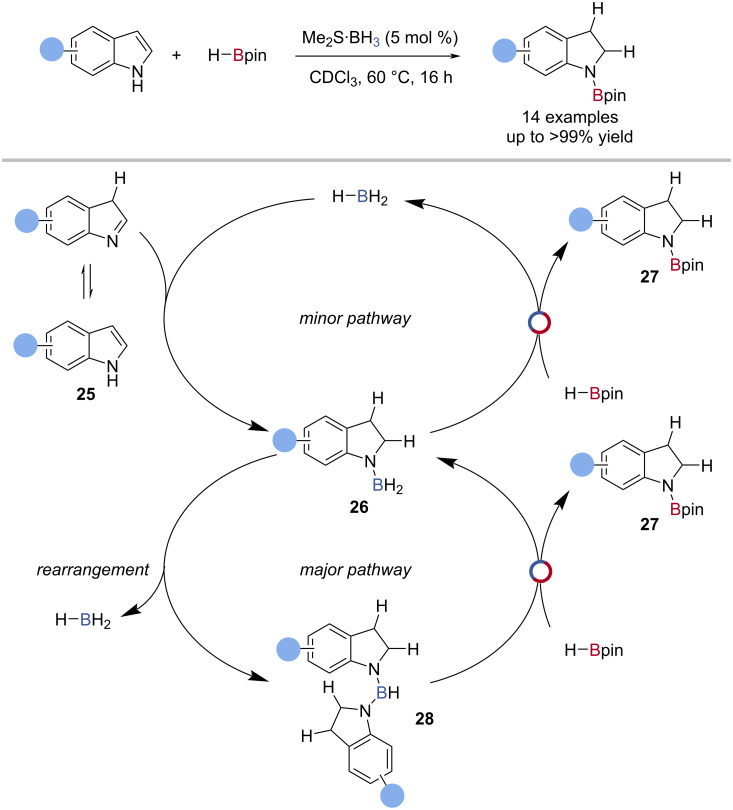
Borane-catalysed reduction of indoles and the proposed mechanism.

The mechanism of stoichiometric indole reduction with Me_2_S·BH_3_ was investigated by Fontaine, and applied to a catalytic variant using HBpin as the turnover reagent (Scheme 7) [70]. Computational analysis showed two plausible, cooperative catalytic cycles: 1) hydroboration of indole **25** with BH_3_ to give a H_2_B-*N*-indoline **26**, which then underwent B‒N/B‒H transborylation with HBpin to regenerate BH_3_ and give the *N*-Bpin-indoline product **27**; 2) two molecules of H_2_B-*N*-indoline underwent rearrangement to regenerate BH_3_ and gave a bisindolinylborane **28**. The bis-*N*-indolinylborane then underwent B‒N/B‒H transborylation with HBpin to regenerate H_2_B-*N*-indoline and gave the Bpin-*N*-indoline product **27**; this was suggested as the major pathway (Scheme 7).

**Scheme 8:**
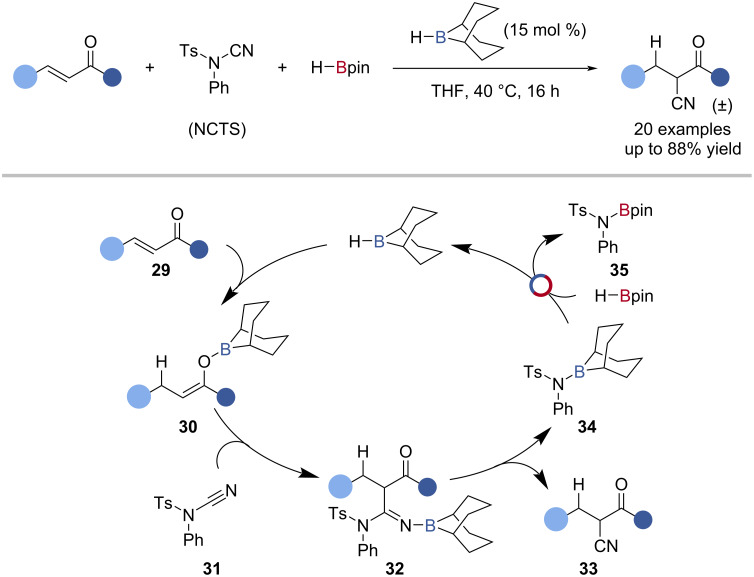
H-*B*-9-BBN-catalysed hydrocyanation of enones and the proposed mechanism.

Thomas et al. reported the H-*B*-9-BBN-catalysed reductive cyanation of enones with HBpin and *N*-cyano-*N*-phenyl-*p*-toluenesulfonamide (NCTS) (Scheme 8) [71]. The reaction was proposed to proceed by 1,4-hydroboration of the enone **29** with H-*B*-9-BBN to give an O-*B*-9-BBN enolate **30**. Electrophilic cyanation of the enolate **30** with NCTS **31**, and elimination gave the β-ketonitrile **33** and TsN(Ph)-9-*B*-BBN **34**, which underwent B‒N/B‒H transborylation with HBpin to regenerate the catalyst and give TsN(Ph)-Bpin **35** (Scheme 8).

**Scheme 9:**
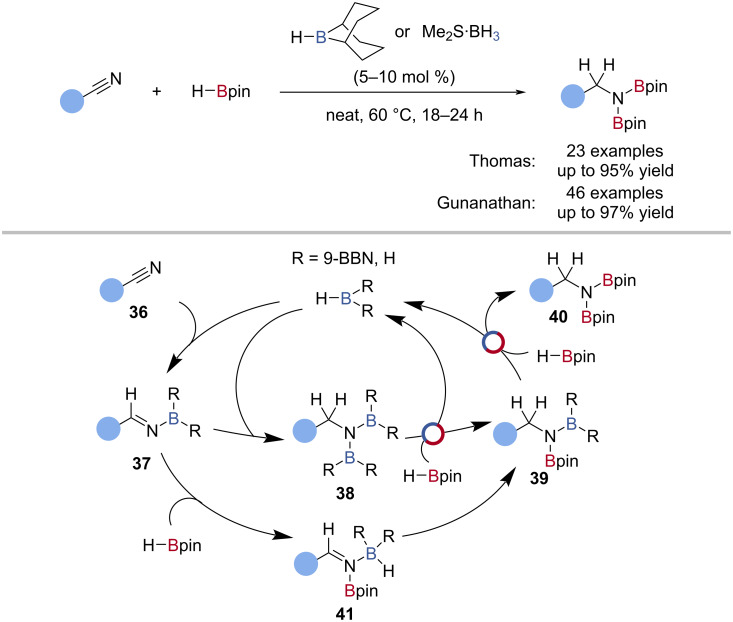
Borane-catalysed hydroboration of nitriles and the proposed mechanism.

Thomas and Gunanathan independently reported the borane-catalysed double hydroboration of nitriles using either Me_2_S·BH_3_ or H-*B*-9-BBN, respectively, as the catalyst and HBpin as the turnover reagent (Scheme 9) [72–73]. Both reports proposed similar mechanisms for the Me_2_S·BH_3_- and H-*B*-9-BBN-catalysed pathways (Scheme 9), where two pathways were operative. The hydroboration of the nitrile **36** gave a borylimine **37**, which underwent a second hydroboration with the borane catalyst to give a diborylamine **38**. Double B‒N/B‒H transborylation of the diborylamine **38** with HBpin regenerated the catalyst and gave the diborylamine **40** product. Alternatively, borylimine **37** underwent a formal B‒N/B‒H transborylation to give a borane-coordinated borylimine-Bpin complex **41**, which underwent hydroboration to give a mixed diborylamine **39**, followed by B‒N/B‒H transborylation to give the diborylamine **40**.

**Scheme 10:**
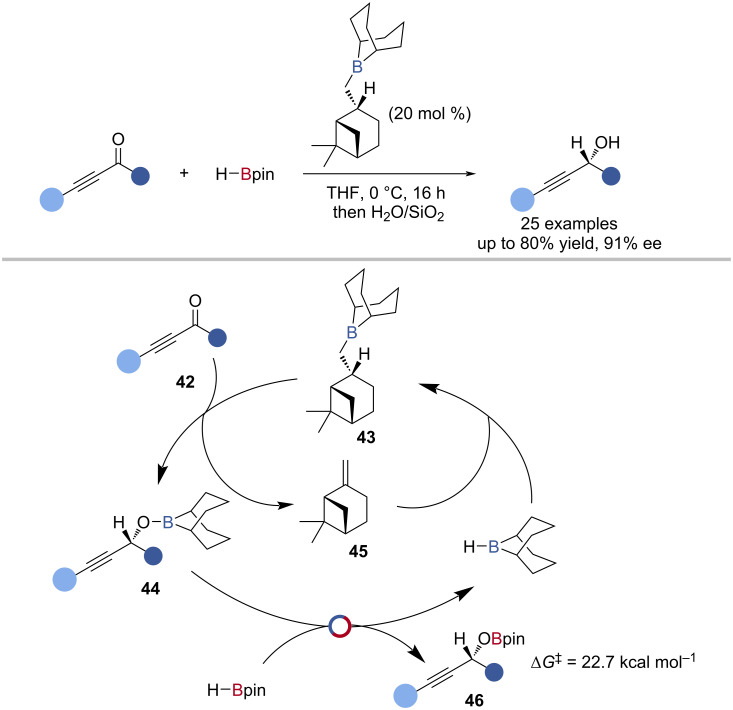
Myrtanylborane-catalysed asymmetric reduction of propargylic ketones and the proposed mechanism.

The first explicit example of a B‒O/B‒H transborylation in catalysis was the catalytic Midland reduction of propargylic ketones developed by Thomas to give enantioenriched propargylic alcohols (Scheme 10) [74]. The reaction was proposed to occur by enantioselective reduction of the propargylic ketone **42** by myrtanyl borane **43** to give an enantioenriched borinic ester **44** and β-pinene **45**. The borinic ester **44** underwent B‒O/B‒H transborylation (Δ*G*^‡^_exp_ = 22.7 kcal mol^−1^) with HBpin giving H-*B*-9-BBN and the alkoxy boronic ester **46**. Hydroboration of β-pinene **45** by H-*B*-9-BBN regenerated the myrtanylborane **43** catalyst. Reaction of H^10^Bpin with the borinic ester intermediate **44** showed no scrambling of the ^10^B label, suggesting a σ-bond metathesis mechanism for this transborylation reaction (Scheme 10).

**Scheme 11:**
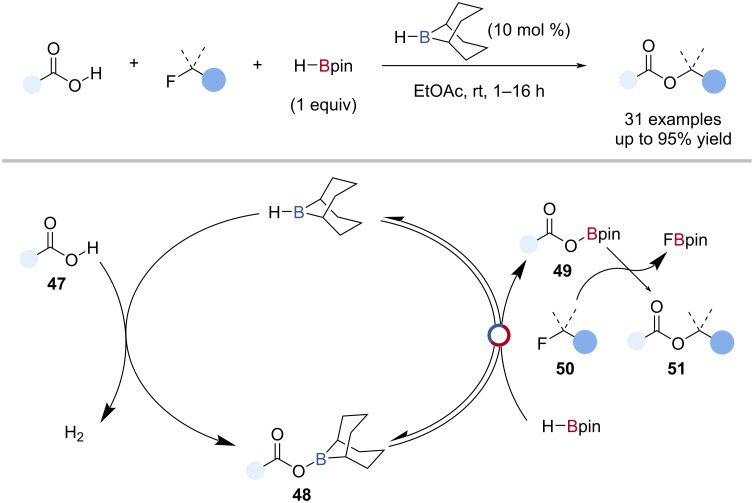
H-*B*-9-BBN-catalysed C–F esterification of alkyl fluorides and the proposed mechanism.

Thomas et al. reported the H-*B*-9-BBN-catalysed esterification of alkyl fluorides, using carboxylic acids and HBpin (Scheme 11) [75]. Through a series of single-turnover experiments a reaction mechanism was proposed where H-*B*-9-BBN catalysed the dehydrocoupling of carboxylic acids **47** with HBpin through B‒O/B‒H transborylation, to give the acyloxy boronic ester **49**. This underwent direct defluoronative carboxylation with the alkyl fluoride **50** to give the ester **51** and FBpin (Scheme 11).

**Scheme 12:**
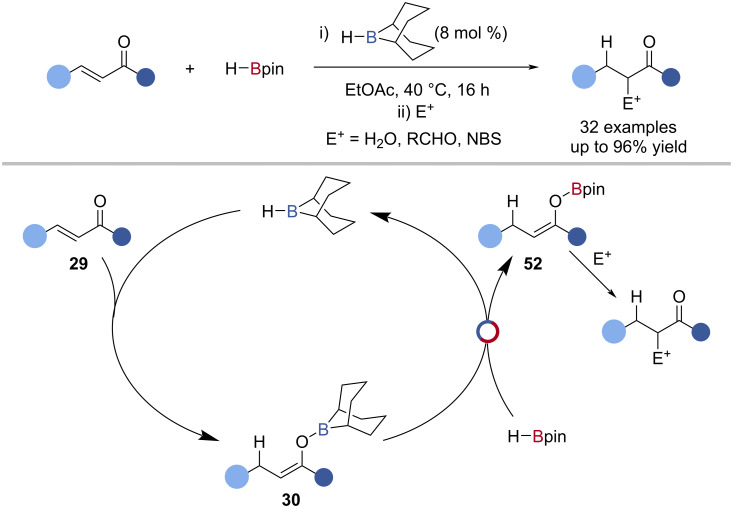
H-*B*-9-BBN-catalysed 1,4-hydroboration of enones and the proposed mechanism.

The H-*B*-9-BBN-catalysed 1,4-hydroboration of enones with HBpin was shown by Thomas, including the subsequent functionalisation of the intermediate Bpin-enolate **52** (Scheme 12) [76]. The proposed mechanism began by 1,4-hydroboration of the enone **29** with H-*B*-9-BBN, followed by B‒O/B‒H transborylation with HBpin to give the Bpin-enolate **52** and regenerate H-*B*-9-BBN (Scheme 12). Isotopic labelling with DBpin and H^10^Bpin supported this proposal.

**Scheme 13:**
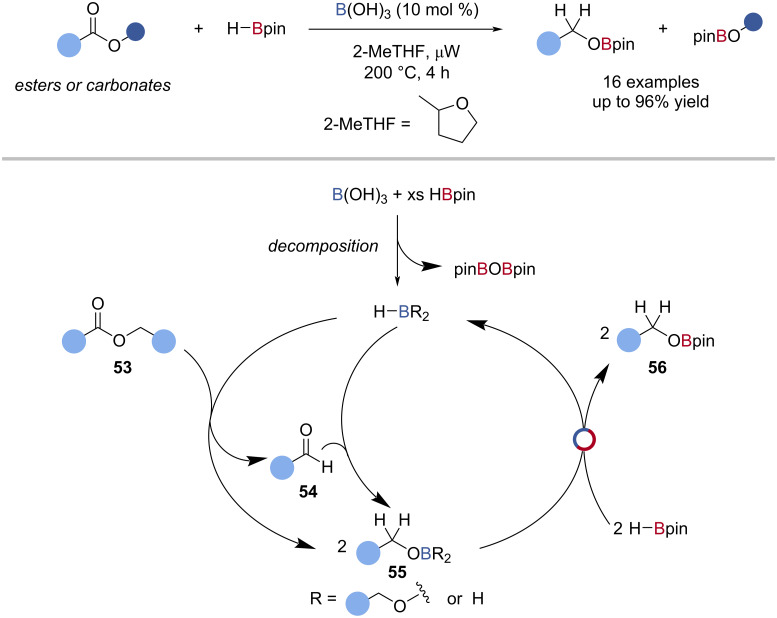
Boric acid-promoted reduction of esters, lactones, and carbonates and the proposed mechanism.

Fontaine reported that boric acid could be used as a precatalyst for the BH_3_-catalysed hydroboration of esters, lactones, and carbonates with HBpin under microwave irradiation (Scheme 13) [57]. When HBpin and boric acid were reacted together, BH_3_-coordinated HBpin and O(Bpin)_2_ were detected by ^11^B NMR spectroscopy. Supported by computational analysis and single-turnover experiments, the reaction was proposed to occur by hydroboration of the carbonyl compound **53** with BH_3_, followed by B‒O/B‒H transborylation with HBpin (Δ*G*^‡^ = 24.5 kcal mol^−1^), to give the alkoxy boronic ester **56** (Scheme 13).

**Scheme 14:**
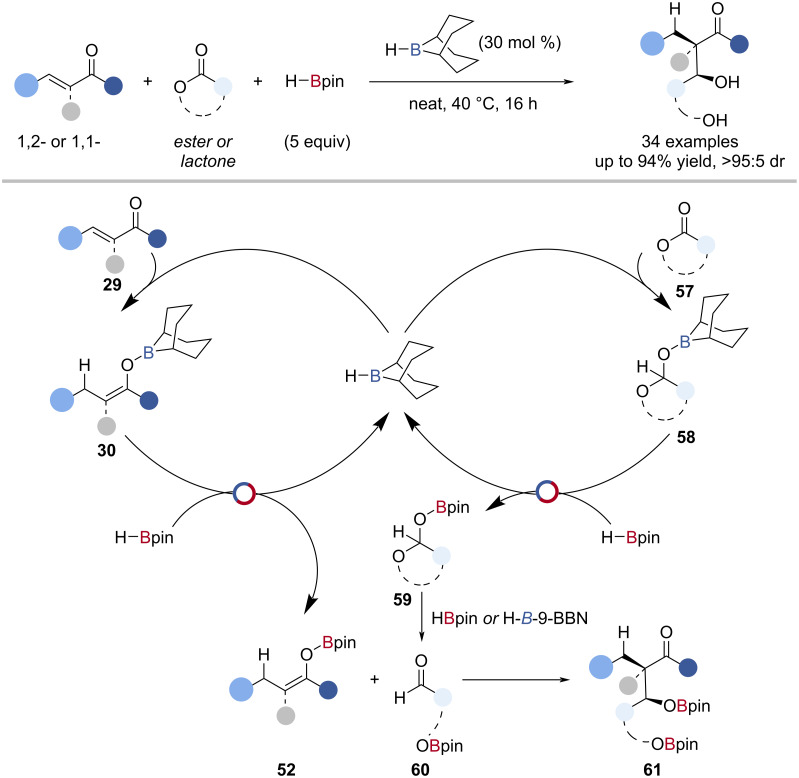
H-*B*-9-BBN-catalysed reductive aldol-type reaction and the proposed mechanism.

Nicholson, Thomas and co-workers reported the H-*B*-9-BBN-catalysed diastereoselective reductive aldol-type reaction of enones and esters or lactones (Scheme 14) [77]. Through a series of single-turnover reactions, a mechanism was proposed (Scheme 14): H-*B*-9-BBN underwent 1,4-hydroboration with the enone **29**, followed by B‒O/B‒H transborylation with HBpin to give an *O*-Bpin enolate **52** and regenerate H-*B*-9-BBN. Alongside this, H-*B*-9-BBN underwent reduction of the ester or lactone **57**, to give a hemiacetal intermediate **58**, which underwent B‒O/B‒H transborylation with HBpin to give an *O*-Bpin hemiacetal **59**. Borane-mediated collapse of the *O*-Bpin hemiacetal gave an aldehyde **60** which reacted with the *O*-Bpin enolate **52** to give aldol-type products **61**.

**Scheme 15:**
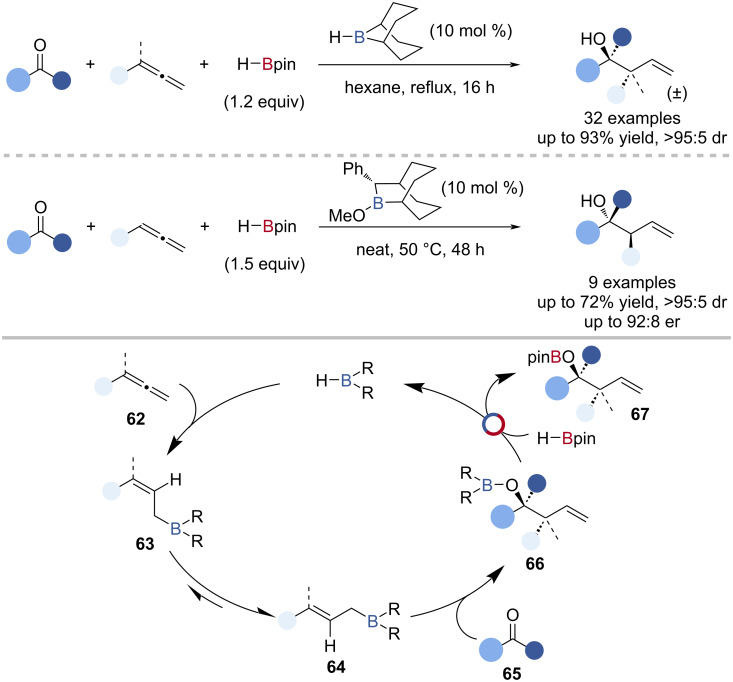
H-*B*-9-BBN-catalysed diastereoselective allylation of ketones and the Ph-BBD-catalysed enantioselective allylation of ketones and the proposed mechanism.

Thomas reported the borane-catalysed diastereo- and enantioselective allylation of ketones with allenes and HBpin to give diastereo- and enantioenriched allylic alcohols, after workup (Scheme 15) [78]. The mechanism was investigated by single-turnover experiments and isotopic labelling and proposed to proceed by hydroboration of the allene **62** by the borane catalyst (H-*B*-9-BBN or 10-phenyl-9-borabicyclo[3.3.2]decane [Ph-BBD]) followed by rapid isomerisation from the (*Z*)-**63** to (*E*)-allylborane **64** which underwent allylation of the ketone **65** to give an allylic borinic ester **66**. B‒O/B‒H transborylation with HBpin gave the *O*-Bpin-protected allylic alcohol **67** and regenerated the borane catalyst (Scheme 15).

**Scheme 16:**
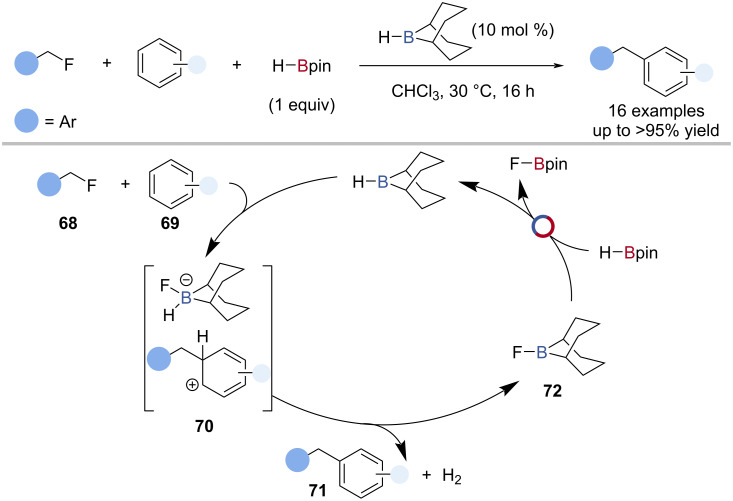
H-*B*-9-BBN-catalysed C–F arylation of benzyl fluorides and the proposed mechanism.

The only example of a B‒F/B‒H transborylation in catalysis comes from Willcox, Thomas and co-workers in the H-*B*-9-BBN-catalysed arylation of alkyl fluorides with HBpin (Scheme 16) [75]. The reaction was proposed to occur through activation of the alkyl fluoride **68** with H-*B*-9-BBN, followed by electrophilic substitution of the arene **69** to give a Wheland intermediate and a fluoroborohydride **70** (Scheme 16). Loss of H_2_ gave the arylated product **71**, dihydrogen, and F-*B*-9-BBN **72**, which underwent B‒F/B‒H transborylation with HBpin, to give FBpin and regenerate H-*B*-9-BBN. The mechanism was confirmed through single-turnover experiments and computational analysis.

**Scheme 17:**
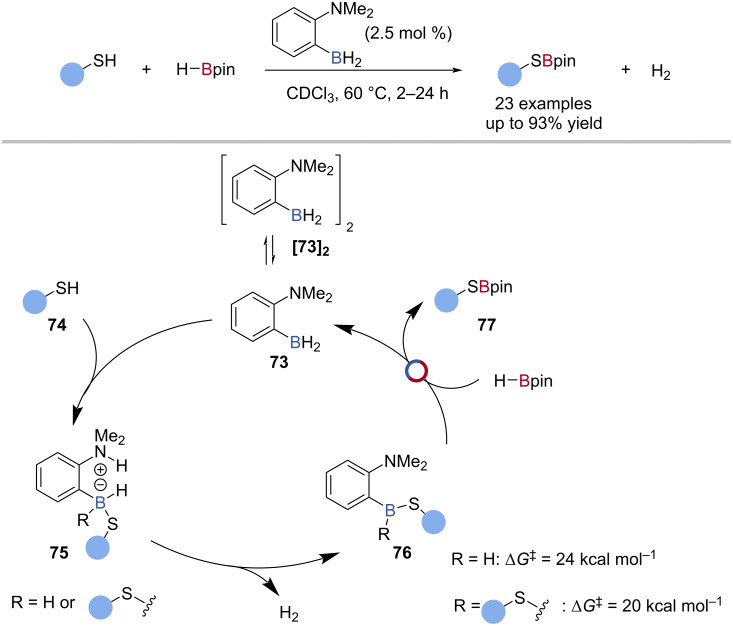
Borane-catalysed S‒H borylation of thiols and the proposed mechanism.

B‒S/B‒H Transborylation in catalysis is also limited to a single example in Fontaine’s FLP-catalysed S‒H borylation of thiols with HBpin (Scheme 17) [79]. Through computational analysis, a mechanism was proposed whereby the ambiphilic amine-borane **73** underwent concerted addition to the thiol **74** S–H bond, to give a zwitterion **75**. After loss of H_2_, a neutral thioborane **76** was generated, which underwent B‒S/B‒H transborylation with HBpin, to give the thioBpin **77** product and regenerate the amine-borane catalyst **73** (Scheme 17).

**Scheme 18:**
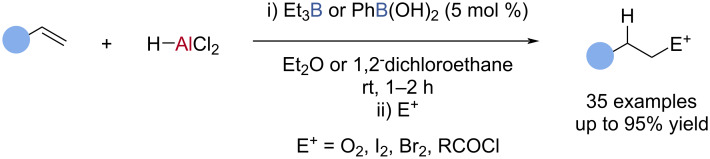
Borane-catalysed hydroalumination of alkenes and allenes.

Yamamoto reported the borane-catalysed hydroalumination of alkenes and allenes (Scheme 18) [80–83] in which the organoaluminium products were reacted in situ with various electrophiles to give formal hydrofunctionalisation products (Scheme 18) [80–83]. Although no mechanism was proposed, a rare B‒C/Al‒H exchange may be responsible for catalytic turnover.

### Aluminium catalysis

**Scheme 19:**
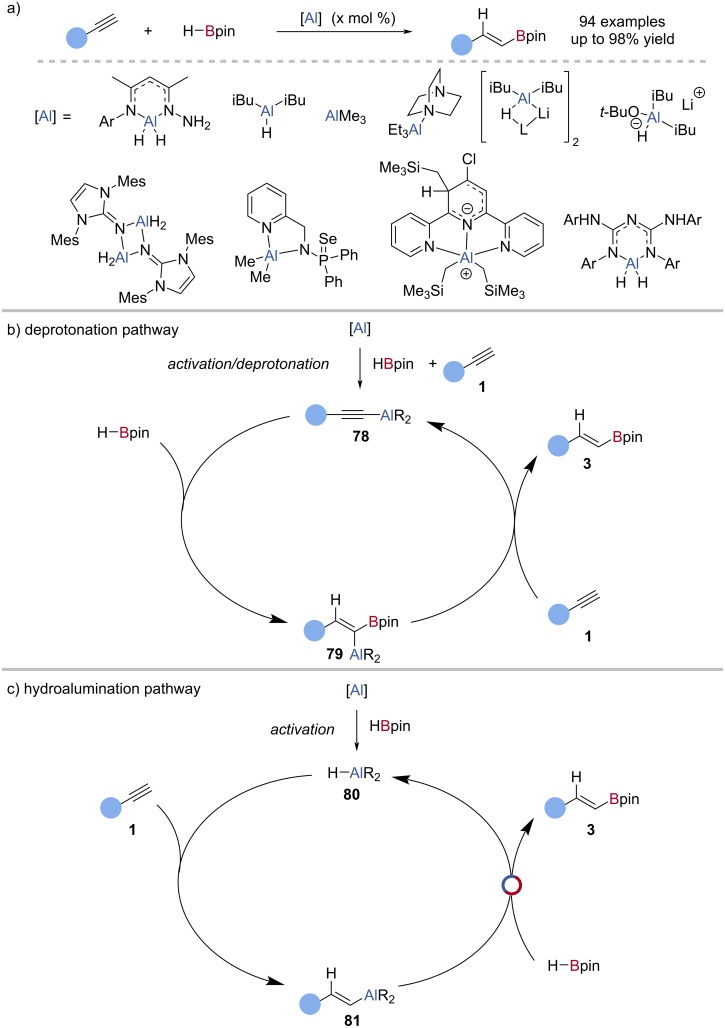
a) Aluminium-catalysed hydroboration of alkynes and example catalysts. b) Deprotonation mechanistic proposal. c) Hydroalumination mechanistic proposal.

The aluminium-catalysed hydroboration of alkynes with HBpin has been well studied with a variety of aluminium complexes having been shown to be catalytically active (Scheme 19a) [84–93]. Roesky reported the first example, using an *N*,*N*′-bis-2,6-diisopropylphenyl diketiminate (NacNac)-supported aluminium dihydride complex as the catalyst [84]. Through computational analysis, the mechanism was proposed to occur by dehydrocoupling between the aluminium dihydride and the alkyne **1** to give an alkynylaluminium species **78**. Direct hydroboration of the alkynyl aluminium species by HBpin gave a *gem*-aluminyl-boryl-alkene **80** which underwent selective protodemetallation with another molecule of alkyne **1** to give the alkenylboronic ester **3** and regenerate an alkynylaluminium species **78** (Scheme 19b). Thomas et al. proposed a different mechanism for the diisobutylaluminium hydride (DIBALH)- or Et_3_Al·DABCO-catalysed hydroboration of alkynes [86], whereby an aluminium hydride **81** underwent hydroalumination of the alkyne **1**, followed by Al‒C/B‒H exchange with HBpin, to give the alkenylboronic ester **3** and regenerate the aluminium hydride **81** (Scheme 19c). Single-turnover experiments and a lack of observable H_2_ production supported this hypothesis. It should also be noted that nucleophilic bases, including LiAlH_4_, promoted the decomposition of HBpin to BH_3_ which can mediate hidden catalysis [56].

**Scheme 20:**
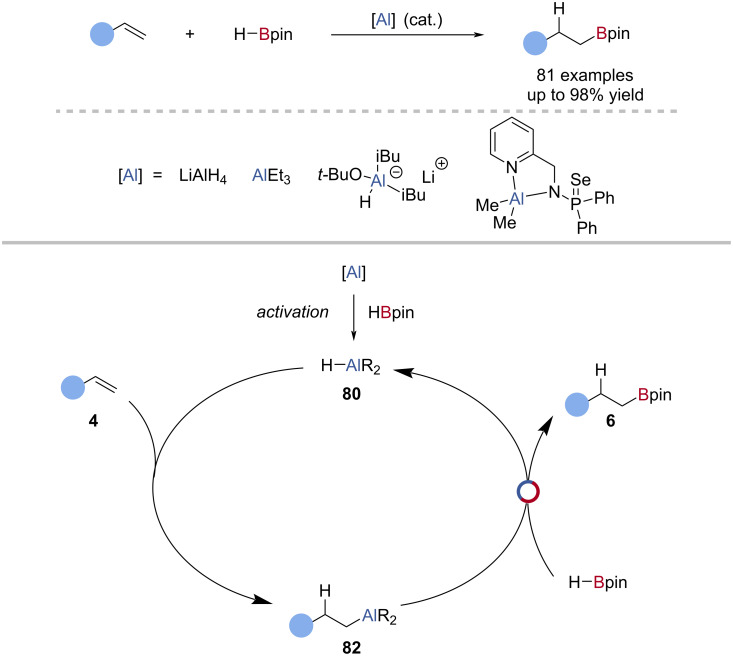
Aluminium-catalysed hydroboration of alkenes and the proposed mechanism.

Thomas et al. reported the aluminium-catalysed hydroboration of alkenes, using HBpin and LiAlH_4_ as the catalyst (Scheme 20) [94]. Through single-turnover experiments they suggested a mechanism similar to aluminium-catalysed alkyne hydroboration; hydroalumination of the alkene **4** by the alane catalyst **80**, Al‒C/B‒H exchange with HBpin, to give the alkylboronic ester **6** and regenerate the alane catalyst **80**. A hydride-mediated decomposition of HBpin and hidden catalysis were not ruled out, as the use of LiH or NaH in place of LiAlH_4_ gave moderate yields of the hydroboration product, however, comparison of the rates of reaction showed the aluminium had an active catalytic role (Scheme 20) [56]. Shi et al. reported that triethylaluminium catalysed the hydroboration of alkenes, under similar conditions to those of Thomas et al. [91]. Other ligand frameworks and aluminate species have shown competence for aluminium-catalysed alkene hydroboration [92,95], with Panda reporting the only reaction which proceeded at room temperature reaction using [κ^2^-{Ph_2_P(Se)NCH_2_(C_5_H_4_N)}Al(CH_3_)_2_] as the catalyst [90].

**Scheme 21:**
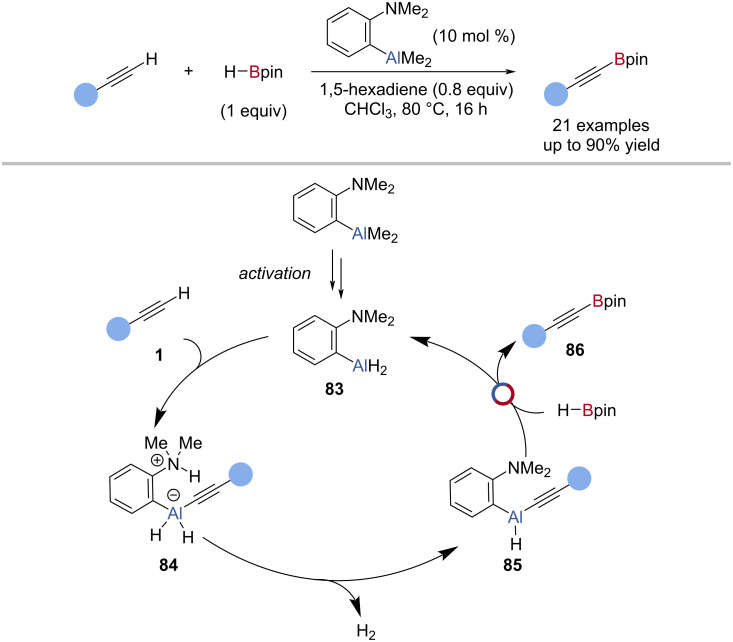
Aluminium-catalysed C–H borylation of terminal alkynes and the proposed mechanism.

Using an ambiphilic aluminium precatalyst, (Me_2_N)C_6_H_4_AlMe_2_, Thomas et al. were able to shut down hydroalumination by the alane and catalyse the C–H borylation of terminal alkynes with HBpin (Scheme 21) [96]. Through kinetic analysis, it was found that the rate of the alkynyl-Bpin product formation was fastest during catalyst activation, rather than during catalysis, leading to an in-depth investigation of catalyst activation using variable time normalisation analysis (VTNA) and kinetic isotope effects. A catalytic cycle was proposed in which (Me_2_N)C_6_H_4_AlH_2_ **83** underwent deprotonation of the alkyne **1** to give a zwitterion **84**. Loss of dihydrogen gave an alkynylaluminium species **85** which underwent Al‒C/B‒H exchange with HBpin to give an alkynylboronic ester **86** and regenerate the catalyst **83** (Scheme 21).

**Scheme 22:**
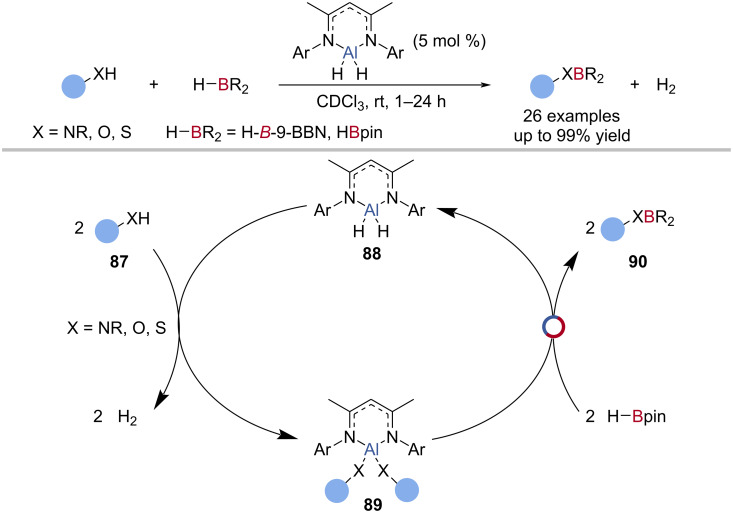
Aluminium-catalysed dehydrocoupling of amines, alcohols, and thiols with H-*B*-9-BBN or HBpin and the proposed mechanism.

Roesky reported the first example of Al‒N/B‒H exchange in catalysis: a NacNac-supported aluminium dihydride-catalysed the dehydrocoupling of HBpin or H-*B*-9-BBN with primary and secondary amines (Scheme 22) [84]. The reaction was proposed to proceed by double dehydrocoupling of the amine **87** and aluminium dihydride **88** to give a bisamido aluminium species **89** which underwent Al‒N/B‒H exchange with HBpin to give the borylated amide **90** and regenerate the aluminium hydride **88** (Scheme 22). This method was also applied to the dehydrocoupling of alcohols and thiols, with this being the only example of an Al‒S/B‒H exchange in catalysis.

**Scheme 23:**
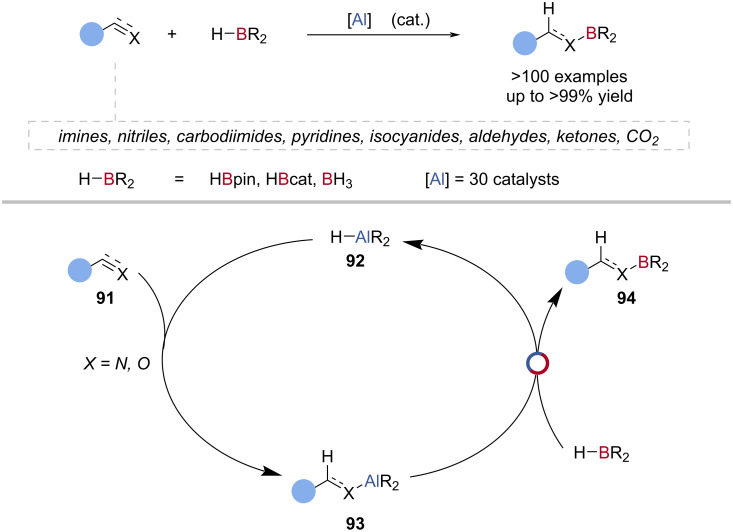
Aluminium-catalysed hydroboration of unsaturated compounds and the general reaction mechanism.

A number of aluminium hydride species has been used for the catalytic hydroboration of imines [87,92,97], nitriles [92,98–101], carbodiimides [92,100,102], pyridine [92], and isocyanides [92] with HBpin (Scheme 23). These generally follow a similar proposed catalytic cycle; aluminium-mediated reduction, followed by Al‒N/B‒H exchange with HBpin (Scheme 23).

The first example of Al‒O/B‒H exchange in catalysis was reported by Woodward, in the enantioselective catalytic hydroboration of ketones with HBcat as the terminal reductant (Scheme 23) [103]. A mixture of 1,1′-bi-2-naphthol (BINOL), 1,1'-binaphthalene-2,2'-dithiol (DTBH_2_), or 2-hydroxy-2'-mercapto-1,1'-binaphthyl (MTBH_2_) with LiAlH_4_ as the catalyst gave good yields of the alcohol (70–80%) after workup, but in low enantioselectivities (1–6% ee). A mechanism was proposed whereby reduction of the ketone **91** by the aluminium hydride **92** was followed by Al‒O/B‒H exchange with HBcat (Scheme 23).

Roesky reported the aluminium-catalysed reduction of aldehydes and ketones with HBpin, using a NacNac-supported aluminium hydride catalyst (Scheme 23) [104]. Using computational analysis, the reaction was proposed to proceed through reduction of the carbonyl **91** by the aluminium hydride **92**, to give an alkoxy aluminium species **93**, followed by Al‒O/B‒H exchange with HBpin to give the alkoxy boronic ester **94** and regenerate the aluminium hydride **92** (Scheme 23). Several aluminium hydride compounds have been reported as competent carbonyl hydroboration catalysts, with proposed mechanisms similar to Roesky’s initial report [85,88–89,97,101,105–109]. The aluminium-catalysed hydroboration of CO_2_ was reported by Fontaine using tris(triphenylphosphine)aluminium as the catalyst and HBcat as the terminal reductant [110]. So et al. reported the bis(phosphoranyl)methanido aluminium hydride-catalysed reduction of CO_2_ with Me_2_S·BH_3_ as the terminal reductant [101].

### Gallium catalysis

**Scheme 24:**
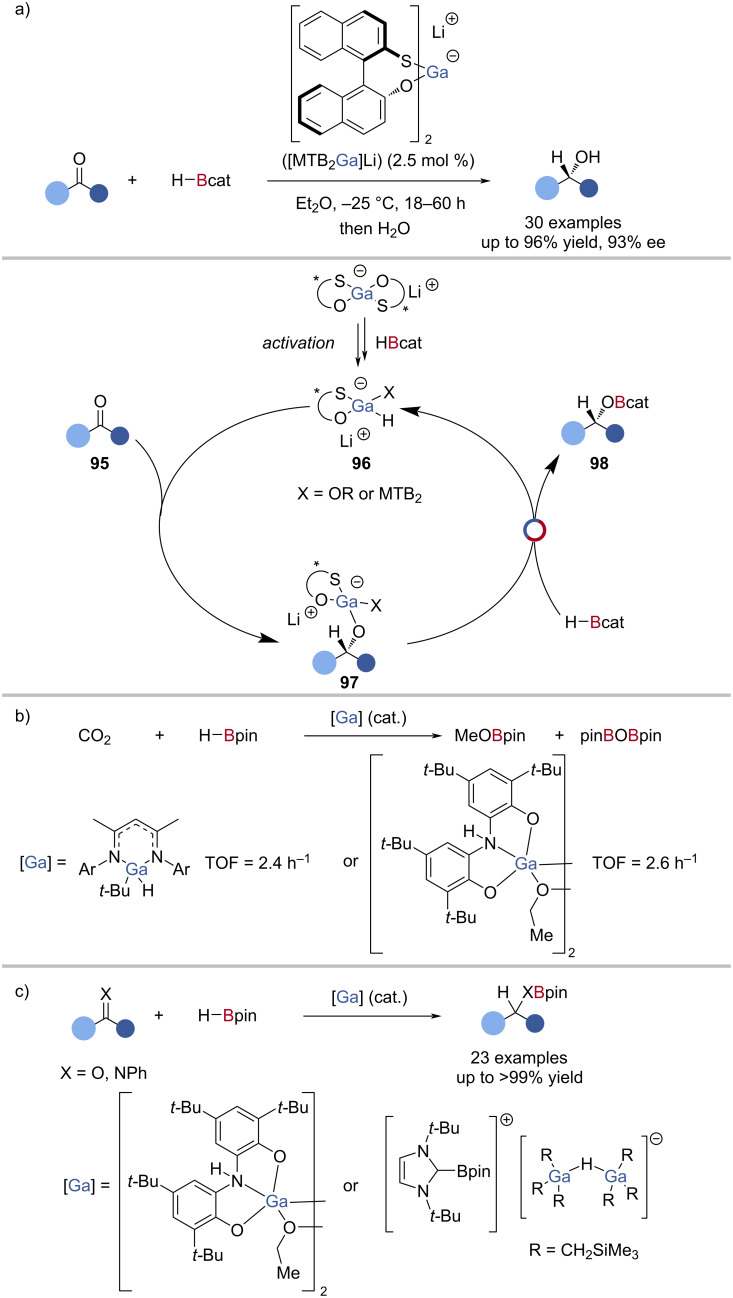
a) Gallium-catalysed asymmetric hydroboration of ketones and the proposed mechanism. b) Gallium-catalysed hydroboration of CO_2_. c) Gallium-catalysed hydroboration of ketones and imines.

Pioneering studies by Woodward reported the enantioselective reduction of ketones using HBcat and a mixture of MTBH_2_/LiGaH_4_ as the catalyst, achieving high yields (up to 96%) and enantioselectivities (up to 93% ee) (Scheme 24a) [111]. The reaction was proposed to proceed through the enantioselective reduction of the ketone **95** by gallium hydride **96**, followed by Ga‒O/B‒H exchange with HBcat to give an enantioenriched alkoxy catechol borane **98**, affording the alcohol after workup (Scheme 24a). The mechanism was later explored in more detail, and the scope expanded, suggesting the reaction proceeded in a similar manner to the Corey–Bakshi–Shibata (CBS) reduction [112], whereby the gallium complex acts as an ambiphilic species coordinated to a ketone, activating it towards reaction with pre-coordinated HBcat [103].

Aldrich expanded the use of gallium in reductive catalysis by showing that a NacNac-supported gallium hydride catalysed the hydroboration of CO_2_ with HBpin to give MeOBpin and O(Bpin)_2_ (Scheme 24b) [113]. Through single-turnover experiments, the gallium hydride was observed to reduce CO_2_ giving a gallium formate complex, which underwent Ga‒O/B‒H exchange with HBpin to afford *O*-Bpin formate and regenerate the gallium hydride. The analogous NacNac-supported aluminium complex was not catalytically competent for the hydroboration of CO_2_, which was rationalised by the unfavourable thermodynamics of the analogous Al‒O/B‒H exchange [114]. Hevia reported a combination of a tris(alkyl)gallium species and bulky *N*-heterocyclic carbene acted as an FLP for B‒H insertion, and was used subsequently as a catalyst in the hydroboration of ketones, aldehydes, esters, and imines with HBpin [115]. Using an ONO-pincer-supported gallium hydride, Goicoechea showed the catalytic hydroboration of ketones and CO_2_ with HBpin. This was also proposed to proceed by carbonyl reduction and Ga‒O/B‒H exchange (Scheme 24c) [116].

**Scheme 25:**
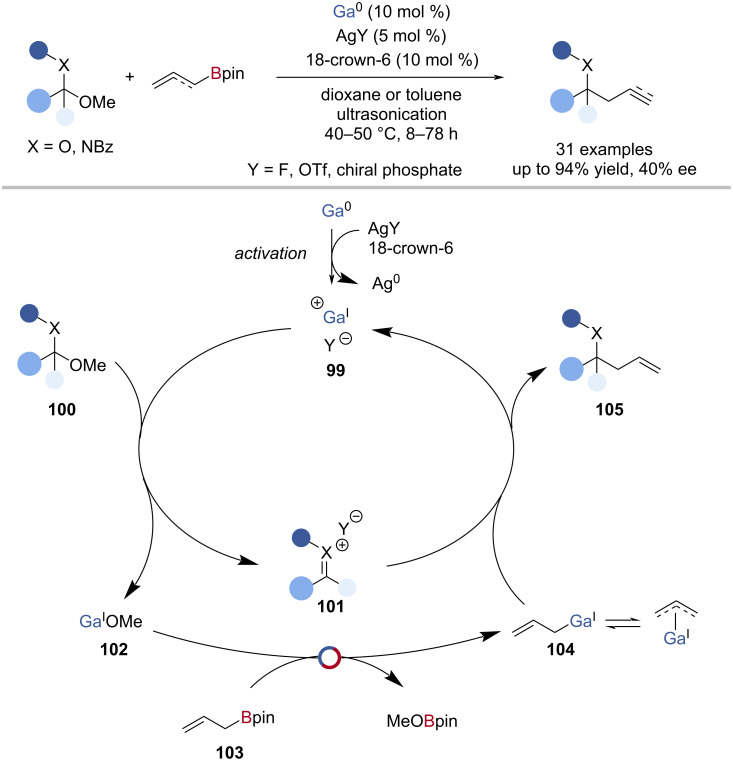
Gallium(I)-catalysed allylation/propargylation of acetals and aminals and the proposed mechanism.

Schneider has shown that a mixture of Ga^0^, AgOTf, and 18-crown-6 catalysed the allylation of acetals, ketals, or aminals with allylic or allenylboronic esters (Scheme 25) [117]. The reaction was proposed to proceed by an activation of elemental gallium to a Ga^I^ species [(18-crown-6)-Ga^I^·(dioxane)*_n_*OTf] **99**, which abstracted methoxide from the acetal **100**, to give an oxocarbenium **101** and Ga^I^OMe **102**. The gallium(I) methoxide (**102**) underwent Ga‒O/B‒C exchange with allyl-Bpin **103** to give MeOBpin and an allylic gallium(I) species **104**, which reacted with the oxocarbenium **103** to give the allylic ether **105** and regenerate the Ga^I^ catalyst **99** (Scheme 25). Using allenylBpin, the selective propargylation of acetals was also achieved. When AgOTf was replaced with silver (*R*)-BINOL phosphate, the asymmetric allylation proceeded in a moderate yield (60%) and enantioselectivity (40% ee). The structure of the ‘Ga^I^OTf’ species was explored in more detail by Slattery, and a monovalent [Ga^I^(18-crown-6)OTf] complex was isolated and characterised by X-ray crystallography, lending support to the mechanism proposed by Schneider [118].

### Indium catalysis

**Scheme 26:**
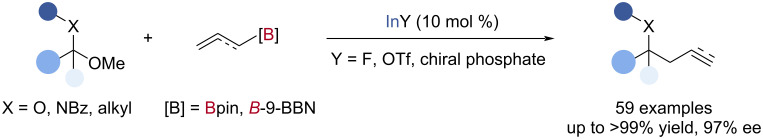
Indium(I)-catalysed allylation/propargylation of acetals, aminals, and alkyl ethers.

Examples of group 13 exchange are limited with indium, even stoichiometrically [36,45], however Kobayashi demonstrated the In^I^-catalysed addition of allylic and allenylboranes to ketals, acetals, aminals, and alkyl ethers (Scheme 26) [119–121]. The proposed mechanism was analogous to the Ga^I^ catalysis by Schneider, with an In‒O/B‒C exchange proposed to drive catalytic turnover.

**Scheme 27:**
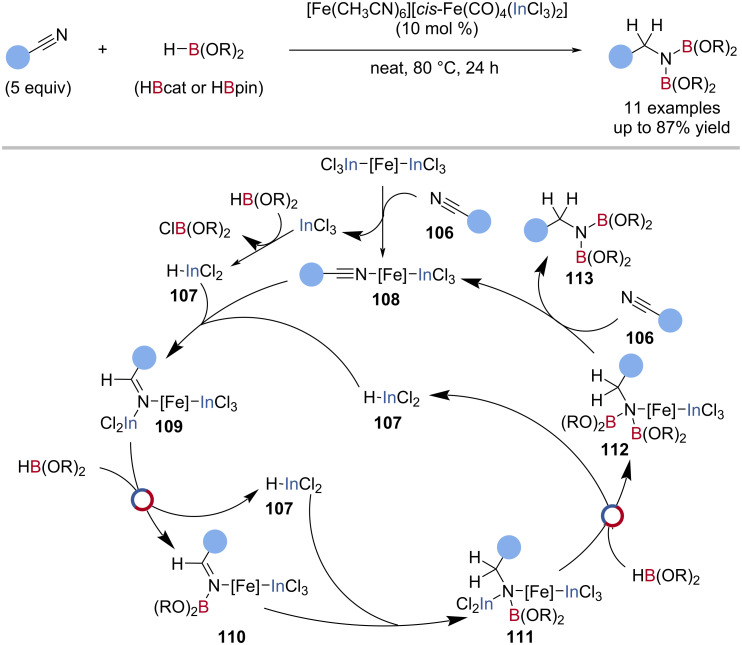
Iron–indium cocatalysed double hydroboration of nitriles and the proposed mechanism.

Nakazawa reported an iron–indium cooperative catalytic system for the hydroboration of nitriles with HBpin and HBcat (Scheme 27) [122]. The precatalyst ([Fe(CH_3_CN)_6_][*cis*-Fe(CO)_4_(InCl_3_)_2_]) was activated in situ with HBpin to give ClBpin and HInCl_2_ **107** by In‒Cl/B‒H exchange. The indium hydride **107** underwent hydroelementation of an iron-coordinated nitrile **108**, to give an indylimine iron complex **109**, which after In‒N/B‒H exchange with HBpin gave a borylimine iron complex **110**. A second hydroelementation and In‒N/B‒H exchange gave the bisborylamine **113** and regenerated the HInCl_2_ **107** co-catalyst (Scheme 27).

## Conclusion

Increasing concerns over the sustainability and toxicity of many transition-metal catalysts has led synthetic chemists to seek alternative elements for catalysis. Group 13 compounds have been at the forefront of chemical research for the past century as organic reagents and functional handles. Group 13 exchange reactions have enabled these reagents to move beyond stoichiometric reactivity to be rendered catalytic, and exhibit catalysis outwith Lewis acid-type activation. These exchange reactions have allowed redox-neutral catalysis complementary to and beyond the redox catalysis of the transition metals.

**Figure 1:**
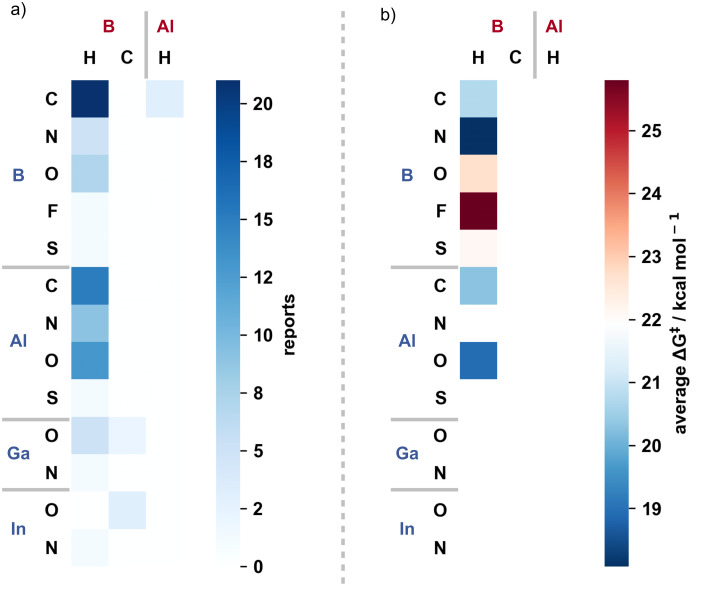
a) The number of reports for a given group 13 exchange in catalysis. b) Average free energy barrier to group 13 exchange in catalysis (blank where no free energy value was reported).

Boron, aluminium, gallium, and indium have all been demonstrated in catalytic transformations using group 13 exchange from alkene functionalisation to carbonyl reduction. The subtle differences in reactivity of the group 13 catalysts were used to enable unique catalytic reactivity and/or reaction chemo- or stereoselectivity, including cases where the stoichiometric reaction was rendered catalytic and, more significantly, where no stoichiometric precedent was known. Group 13 exchange reactions being the driver for new chemical reactivity and unique molecular disconnections. This is not to say that all stoichiometric group 13 reactions have been rendered catalytic, or all new reactivity discovered, leaving an exciting future for main group catalysis underpinned by group 13 exchange and transborylation reactions (Figure 1).
